# Association of Conventional and Electronic Cigarette Use with Obesity and Central Obesity Among Korean Adults: A Cross-Sectional Analysis of the 2016–2018 KNHANES (2016–2018)

**DOI:** 10.3390/medicina62050909

**Published:** 2026-05-08

**Authors:** JooHee Park, Sangwon Hwang, Sung-Kyung Kim, Hoon Jo

**Affiliations:** 1 Department of Physical Therapy, College of Software and Digital Healthcare Convergence, Yonsei University, Wonju 26493, Republic of Korea; pjh1325@naver.com; 2 Department of Precision Medicine, Wonju College of Medicine, Yonsei University, Wonju 26426, Republic of Korea; arsenal@yonsei.ac.kr; 3 Institute of Artificial Intelligence and Big Data in Medicine, Wonju College of Medicine, Yonsei University, Wonju 26426, Republic of Korea; 4 Department of Preventive Medicine, Wonju College of Medicine, Yonsei University, Wonju 26426, Republic of Korea; stacte@yonsei.ac.kr; 5 Department of Dementia Prevention & Rehabilitation, College of Human Service, Catholic Kwandong University, Gangneung 25601, Republic of Korea

**Keywords:** abdominal obesity, cross-sectional studies, electronic nicotine delivery systems, smoking

## Abstract

*Background and Objectives:* This study aimed to examine whether the association between smoking status and both obesity and abdominal obesity extends to electronic cigarette use. *Materials and Methods:* This cross-sectional, nationally representative study used data from the Korea National Health and Nutrition Examination Survey (KNHANES) collected between 2016 and 2018. Smoking status was categorized with consideration of electronic cigarette use. Multivariable logistic regression analyses were performed to examine the associations between smoking status and obesity as well as abdominal obesity. *Results:* After adjusting for sex, age, physical activity, alcohol consumption, hypertension, and diabetes, electronic cigarette users had significantly higher odds of obesity and abdominal obesity than non-smokers (obesity: OR 1.98, 95% CI 1.16–3.38, *p* = 0.013; abdominal obesity: OR 2.03, 95% CI 1.16–3.53, *p* = 0.013). In comparisons among current smokers, dual users had significantly higher odds of obesity and abdominal obesity than conventional cigarette smokers (obesity: OR 1.55, 95% CI 1.23–1.96, *p* < 0.001; abdominal obesity: OR 1.30, 95% CI 1.01–1.68, *p* = 0.040). *Conclusions:* Overall, smoking status was associated with a higher prevalence of abdominal obesity, with particularly elevated risk among electronic cigarette and dual users. While the findings for dual users were robust, those for exclusive electronic cigarette users were based on a small sample and should be interpreted as exploratory. Larger studies are needed to confirm these preliminary observations.

## 1. Introduction

Smoking is a major risk factor for a wide range of chronic diseases and is known to play an important role in body weight regulation by influencing multiple physiological mechanisms, including energy intake, physical activity levels, basal metabolic rate, and inflammatory responses [1,2,3]. Although many epidemiological studies have reported associations between smoking and either weight gain or weight loss [4,5,6], the underlying mechanisms and the direction of these relationships remain inconsistent, and the association between smoking and obesity is still not clearly established. A large-scale epidemiological study conducted in China in 2015 reported that the risk of obesity increased with greater smoking intensity and cumulative exposure [5]. In particular, smoking appears to be more strongly associated with abdominal obesity than with general obesity defined solely by body mass index (BMI) [7,8].

As tobacco products diversify and smoking patterns change, electronic cigarettes (e-cigarettes), a newer form of tobacco use, have rapidly increased. According to the Korea Disease Control and Prevention Agency (KDCA) in 2024, the prevalence of e-cigarette use in Korea increased from 5.1% in 2019 to 8.1% in 2023, and the prevalence among women more than doubled over the same period, rising from 1.0% to 2.1%. Similar increasing trends have also been observed in other countries, including the United States [9].

E-cigarettes deliver nicotine by heating and aerosolizing a liquid solution without combustion. They were originally developed to support smoking cessation and reduce nicotine dependence, and many users believe that e-cigarettes are less addictive and less carcinogenic than conventional cigarettes, may facilitate smoking cessation, and may also help with weight control or stress reduction [10]. Although e-cigarettes were initially considered relatively less harmful because they expose users to fewer combustion-related toxicants such as tar and carbon monoxide, real-world use patterns suggest that they often do not fully replace conventional cigarettes. Instead, concerns have been raised that e-cigarettes may promote dual use, or serve as a gateway to smoking initiation among non-smokers and adolescents [11,12]. In addition, e-cigarettes are frequently marketed with youth-oriented designs and flavored products (e.g., sweet or menthol flavors), often portrayed as safer and more fashionable, which may encourage smoking uptake among young women and adolescents [13,14,15].

Despite the rapid increase in e-cigarette use, evidence regarding its association with weight-related outcomes remains limited and inconsistent. Some studies have suggested that e-cigarette use is more prevalent among individuals who are overweight or obese, whereas others have reported associations with weight loss [6]. Therefore, further research is needed to clarify the relationship between e-cigarette use and obesity-related outcomes.

Therefore, this study aimed to examine the associations between smoking status and obesity as well as abdominal obesity using data from the 7th Korea National Health and Nutrition Examination Survey (KNHANES, 2016–2018). By comparing obesity and abdominal obesity across these smoking patterns, this study seeks to examine the association between smoking patterns and obesity-related outcomes according to smoking type.

## 2. Materials and Methods

### 2.1. Study Population

This study was a cross-sectional analysis using data from the Korea National Health and Nutrition Examination Survey (KNHANES) conducted between 2016 and 2018. KNHANES is a nationwide, population-based survey administered by the Korea Disease Control and Prevention Agency (KDCA) to assess the health and nutritional status of the non-institutionalized Korean population. KNHANES applies a multistage stratified cluster sampling design based on geographic area, sex, and age to ensure national representativeness.

The survey consists of three components: a health interview, a health examination, and a nutrition survey. These components provide comprehensive information on demographic characteristics, health behaviors (including smoking and alcohol consumption), medical history of chronic diseases, anthropometric measurements (e.g., height, weight, and waist circumference), and dietary intake. In this study, data from KNHANES VII (2016–2018), which included information on smoking behaviors—including electronic cigarette use—and anthropometric measurements necessary to assess general and central obesity, were analyzed.

**Figure 1:**
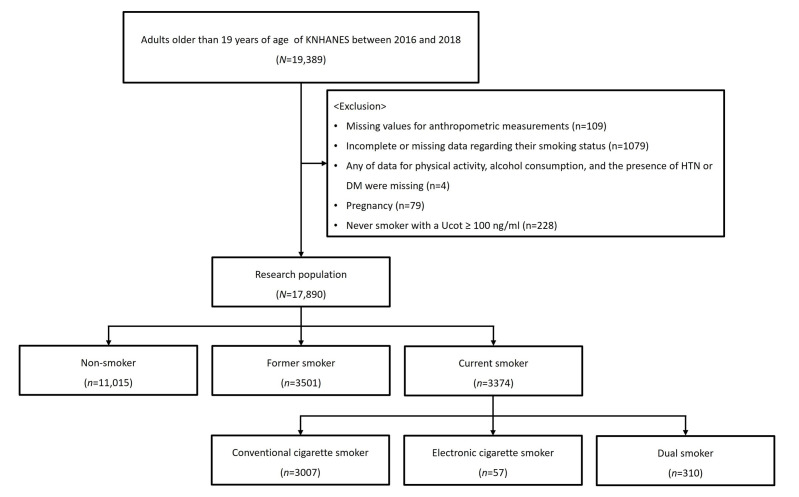
Flowchart of the inclusion and exclusion criteria.

Among the 19,389 participants initially included, 1079 individuals with insufficient information regarding smoking status and 109 individuals with missing anthropometric data were excluded. In addition, 4 participants with missing data on physical activity, alcohol consumption, or diagnosis of hypertension or diabetes were excluded. Pregnant women (*n* = 79) were also excluded. Furthermore, because misreporting of non-smoking status has been reported to be relatively common in East Asian populations, participants with a urinary cotinine concentration ≥ 100 ng/mL (*n* = 228) were excluded [16]. As a result, a total of 17,890 participants were included in the final analysis (Figure 1).

### 2.2. Ethical Approval

KNHANES was conducted with approval from the Institutional Review Board of the Korea Disease Control and Prevention Agency, and the IRB approval number was 2018-01-03-P-A. The dataset used in this study was obtained from the KNHANES website (https://knhanes.cdc.go.kr, accessed on 15 December 2025) after completing the required application procedures. All personal identifiers were removed, and the data were collected using anonymized unique identification numbers to ensure confidentiality. Therefore, this study did not pose any direct harm to participants.

### 2.3. Smoking Status

Smoking status was classified based on self-reported questionnaire responses and urinary cotinine concentrations. Participants were categorized into five groups: non-smokers, former smokers, conventional cigarette smokers, electronic cigarette smokers, and dual smokers. It should be noted that this classification may not fully capture all forms of nicotine product use, as KNHANES VII did not include specific items on heated tobacco products or nicotine replacement therapy.

Non-smokers were defined as individuals who had smoked fewer than 100 cigarettes in their lifetime, had not used electronic cigarettes within the past month, and had a urinary cotinine concentration < 100 ng/mL [17]. Former smokers were defined as individuals who had smoked at least 100 cigarettes in their lifetime but were not currently smoking and had never used electronic cigarettes or had not used them within the past month. Conventional cigarette smokers were defined as individuals who had smoked at least 100 cigarettes in their lifetime, were currently smoking conventional cigarettes, and had never used electronic cigarettes or had not used them within the past month. Electronic cigarette smokers were defined as individuals who had ever used electronic cigarettes and had used them within the past month. Dual smokers were defined as individuals who had smoked at least 100 cigarettes in their lifetime, were currently smoking conventional cigarettes, and had used electronic cigarettes within the past month.

Urinary cotinine is a metabolite of nicotine and is widely used as a biological marker for assessing smoking exposure. In this study, urinary cotinine concentration was measured using high-performance liquid chromatography–tandem mass spectrometry (HPLC-MS/MS) with the Agilent 1100 Series (Agilent Technologies, Santa Clara, CA, USA) and API 4000 system (AB Sciex, Framingham, MA, USA). Cotinine levels were recorded to one decimal place and reported in ng/mL [18].

### 2.4. Obesity

Obesity was defined as a body mass index (BMI) ≥ 25 kg/m^2^ [19]. During the health examination, participants removed their shoes and any carried items before anthropometric measurements were obtained. Height was measured to the nearest 0.1 cm, and body weight was measured to the nearest 0.1 kg. BMI was calculated as weight (kg) divided by height squared (m^2^). Abdominal obesity was defined as a waist circumference ≥ 90 cm in men and ≥85 cm in women [20].

### 2.5. Covariates

Educational attainment was categorized into four groups: elementary school graduate or lower, middle school graduate, high school graduate, and college graduate or higher. Household income was adjusted by calculating the equivalized monthly household income (monthly household income divided by the square root of household size) and then classified into quartiles.

Physical activity, alcohol consumption, and physician-diagnosed hypertension or diabetes were assessed using self-reported questionnaire data. Physical activity was classified using the International Physical Activity Questionnaire–Short Form (IPAQ-SF) and categorized into low, moderate, and high levels. Low physical activity was defined as not meeting the criteria for moderate or high physical activity. Moderate physical activity was defined as engaging in vigorous-intensity activity for at least 20 min per day on ≥3 days per week, or a combination of walking, moderate-intensity, and/or vigorous-intensity activities on ≥5 days per week accumulating at least 600 metabolic equivalent of task (MET)-minutes per week. High physical activity was defined as engaging in vigorous-intensity activity on ≥3 days per week accumulating at least 1500 MET-minutes per week, or a combination of walking, moderate-intensity, and/or vigorous-intensity activities performed daily accumulating at least 3000 MET-minutes per week.

Alcohol consumption frequency was collected via questionnaire based on the average weekly drinking frequency and categorized into four groups: less than once per week, once per week, 2–3 times per week, and ≥4 times per week. Hypertension and diabetes were assessed based on self-reported history of physician diagnosis.

### 2.6. Statistical Analysis

All statistical analyses were performed using SPSS version 25.0 for Windows (IBM Corp., Armonk, NY, USA; 2017), and statistical significance was set at *p* < 0.05.

It should be noted that the analyses were conducted using standard logistic regression procedures without incorporating the KNHANES complex sampling design features (i.e., sampling weights, stratification variables, and primary sampling units). While this approach may underestimate standard errors and produce narrower confidence intervals than would be obtained under design-based analysis, prior methodological research suggests that odds ratio point estimates from logistic regression are generally robust to the omission of survey design features, although the precision of those estimates may be overstated [21].

To describe baseline characteristics across smoking groups (non-smokers, former smokers, current conventional cigarette smokers [current c-smokers], current electronic cigarette smokers [current e-smokers], and dual smokers), continuous variables including age, height, weight, waist circumference, BMI, and urinary cotinine levels were compared using analysis of variance (ANOVA), and categorical variables including sex, physical activity, alcohol consumption, hypertension, and diabetes status were compared using the chi-square test.

Multivariable logistic regression analyses were conducted to evaluate the associations of smoking status with obesity and abdominal obesity. Odds ratios (ORs) were presented for the crude model (unadjusted) and the adjusted model controlling for sex, age, physical activity, alcohol consumption, hypertension, and diabetes.

## 3. Results

### 3.1. General Characteristics

Among the total study population, non-smokers accounted for 11,015 participants (61.6%), former smokers for 3501 participants (19.6%), and current smokers for 3374 participants (18.9%). Among current smokers, conventional cigarette smokers (c-smokers) comprised 3007 participants (89.1%), electronic cigarette smokers (e-smokers) comprised 57 participants (1.7%), and dual smokers comprised 310 participants (9.2%).

The dual smoker group had the lowest mean age among the smoking status groups. The mean age was 51.20 (±17.18) years for non-smokers, 57.14 (±15.35) years for former smokers, 47.56 (±15.06) years for c-smokers, 39.33 (±12.01) years for e-smokers, and 38.86 (±11.95) years for dual smokers, showing a significant difference in mean age across smoking categories (*p* < 0.001).

The proportion of males was highest among former smokers (3053 participants [87.2%]) and lowest among non-smokers (2059 participants [18.7%]) (*p* < 0.001). Height was highest in e-smokers (172.88 ± 8.52 cm) and lowest in non-smokers (159.62 ± 8.68 cm) (*p* < 0.001). BMI was highest in e-smokers (25.50 kg/m^2^) and lowest in non-smokers (23.80 kg/m^2^). Waist circumference was also highest in e-smokers (87.60 cm) and lowest in non-smokers (80.38 cm) (*p* < 0.001). Urinary cotinine concentration was highest in dual smokers (1430.60 ng/mL) and lowest in non-smokers (2.11 ng/mL) (*p* < 0.001) (Table 1).

**Table 1:** General characteristics of the subjects.

	Smoking Status					*p*-Value
	Never Smoker	Ex-Smoker	Current Smoker (Total)			
			Current Smoker (c-Smoker)	Current Smoker (e-Smoker)	Current Smoker (Dual Smoker)	
Total number	11,015	3501	3007	57	310	
Age (year)	51.20 ± 17.18	57.14 ± 15.35	47.56 ± 15.06	39.33 ± 12.01	38.86 ± 11.95	<0.001
Male sex	2059 (18.7)	3053 (87.2)	2520 (83.8)	48 (84.2)	268 (86.5)	<0.001
Education ^a^						<0.001
≤Elementary school	2505 (23.6)	652 (19.5)	382 (13.4)	2 (3.6)	9 (3.0)	
Middle school	989 (9.3)	435 (13.0)	312 (11.0)	4 (7.3)	13 (4.3)	
High school	3184 (30.0)	1007 (30.1)	1146 (40.3)	15 (27.3)	130 (43.5)	
≥University	3937 (37.1)	1254 (37.5)	1002 (35.3)	34 (61.8)	147 (49.2)	
House income ^a^						<0.001
Low	2167 (19.7)	712 (20.4)	543 (18.1)	8 (14.0)	23 (7.4)	
Low-middle	2617 (23.8)	873 (25.0)	766 (25.6)	12 (21.1)	70 (22.6)	
Middle-High	2955 (26.9)	908 (26.0)	882 (29.4)	18 (31.6)	101 (32.6)	
High	3242 (29.5)	998 (28.6)	805 (26.9)	19 (33.3)	116 (37.4)	
Alcohol consumption (times/wk)						<0.001
<1	7497 (68.1)	1389 (39.7)	929 (30.9)	17 (29.8)	76 (24.5)	
1	2278 (20.7)	866 (24.7)	718 (23.9)	19 (33.3)	102 (32.9)	
2–3	984 (8.9)	786 (22.5)	858 (28.5)	15 (26.3)	88 (28.4)	
≥4	256 (2.3)	460 (13.1)	502 (16.7)	6 (10.5)	44 (14.2)	
Physical activity						<0.001
Low	5636 (51.2)	1846 (52.7)	1668 (55.5)	23 (40.4)	139 (44.8)	
Moderate	4255 (38.6)	1233 (35.2)	994 (33.1)	26 (45.6)	125 (40.3)	
High	1124 (10.2)	422 (12.1)	345 (11.5)	8 (14.0)	46 (14.8)	
Height (cm)	159.62 ± 8.68	167.81 ± 7.32	169.02 ± 8.07	172.88 ± 8.52	171.79 ± 7.50	<0.001
Weight (kg)	60.58 ± 11.23	69.10 ± 11.46	69.23 ± 12.92	76.85 ± 16.96	74.10 ± 13.63	<0.001
BMI (kg/m^2^)	23.80 ± 3.58	24.51 ± 3.18	24.14 ± 3.66	25.50 ± 4.13	25.07 ± 3.62	<0.001
WC (cm)	80.38 ± 10.10	86.42 ± 9.08	84.77 ± 9.93	87.60 ± 11.80	86.40 ± 9.85	<0.001
Urine cotinine (ng/mL) ^a^	2.11 ± 8.18	61.97 ± 269.47	1264.10 ± 831.89	1258.23 ± 1102.17	1043.60 ± 888.79	<0.001
Underlying disease						
Hypertension	2552 (23.2)	1131 (32.3)	625 (20.8)	8 (14.0)	39 (12.6)	<0.001
Diabetes	957 (8.7)	475 (13.6)	279 (9.3)	5 (8.8)	13 (4.2)	<0.001

Values are presented as mean ± SD or number (proportions). BMI, body mass index; WC, waist circumference; SD, standard deviation; c-smoker, conventional cigarette smoker; e-smoker, electronic cigarette smoker. ^a^ Missing data were excluded from analysis.

### 3.2. Association Between Smoking Status and Obesity

#### 3.2.1. Distribution of Obesity and Abdominal Obesity

The prevalence of obesity and abdominal obesity according to smoking status is presented in Table 2. Obesity prevalence was highest among former smokers (1401 participants [40.0%]) and lowest among non-smokers (3537 participants [32.1%]) (*p* < 0.001). The prevalence of abdominal obesity was also highest among former smokers (1226 participants [35.0%]) and lowest among non-smokers (3067 participants [27.8%]) (*p* < 0.001).

#### 3.2.2. Risk of Obesity

As shown in Table 3, multivariable logistic regression analysis in the crude model demonstrated that current smokers had higher odds of obesity than non-smokers (odds ratio [OR] 1.37; 95% confidence interval [CI], 1.26–1.48). Former smokers also had higher odds of obesity compared with non-smokers (OR 1.41; 95% CI, 1.30–1.53). In the adjusted model controlling for sex, age, physical activity, alcohol consumption, hypertension, and diabetes, current smokers still showed higher odds of obesity than non-smokers; however, the association did not reach statistical significance (OR 1.06; 95% CI, 0.96–1.17).

#### 3.2.3. Risk of Abdominal Obesity

In the crude model, current smokers had significantly higher odds of abdominal obesity than non-smokers (OR 1.24; 95% CI, 1.14–1.34; *p* < 0.001). Former smokers also had significantly higher odds of abdominal obesity compared with non-smokers (OR 1.40; 95% CI, 1.29–1.51; *p* < 0.001). In the adjusted model, current smoking remained significantly associated with higher odds of abdominal obesity compared with non-smoking (OR 1.17; 95% CI, 1.05–1.30; *p* = 0.005). Former smoking was also associated with higher odds of abdominal obesity compared with non-smoking (OR 1.04; 95% CI, 0.93–1.15), but this association was not statistically significant after adjustment (*p* = 0.510).

### 3.3. Association Between Cigarette Type and Obesity Among Current Smokers

#### 3.3.1. Distribution of Obesity and Abdominal Obesity by Cigarette Type

When current smokers were categorized into c-smokers, e-smokers, and dual smokers, obesity prevalence was highest among e-smokers (30 participants [52.6%]) and lowest among c-smokers (1143 participants [38.0%]) (*p* < 0.001). Abdominal obesity prevalence was also highest among e-smokers (23 participants [40.4%]) and lowest among c-smokers (954 participants [31.7%]), although the difference was not statistically significant (*p* = 0.121).

#### 3.3.2. Risk of Obesity by Cigarette Type

In the crude model, c-smokers had higher odds of obesity than non-smokers (OR 1.30; 95% CI, 1.19–1.41) (Table 3). E-smokers (OR 2.35; 95% CI, 1.40–3.96) and dual smokers (OR 2.01; 95% CI, 1.60–2.52) also had higher odds of obesity than non-smokers. The OR was highest among e-smokers and lowest among c-smokers, and these associations were statistically significant (*p* < 0.001). In the adjusted model, e-smokers continued to show significantly higher odds of obesity compared with non-smokers (OR 1.98; 95% CI, 1.16–3.38; *p* = 0.013).

In comparisons among current smokers, e-smokers had significantly higher odds of obesity than c-smokers in the crude model (OR 1.81; 95% CI, 1.07–3.06; *p* = 0.027). However, this association was no longer statistically significant in the adjusted model (OR 1.65; 95% CI, 0.96–2.81; *p* = 0.068). Dual smokers also had significantly higher odds of obesity than c-smokers (OR 1.55; 95% CI, 1.23–1.96; *p* < 0.001). The OR was higher in e-smokers than in dual smokers.

#### 3.3.3. Risk of Abdominal Obesity by Cigarette Type

In the crude model, c-smokers had significantly higher odds of abdominal obesity than non-smokers (OR 1.20; 95% CI, 1.10–1.31; *p* < 0.001) (Table 3). E-smokers (OR 1.75; 95% CI, 1.03–2.98; *p* = 0.038) and dual smokers (OR 1.47; 95% CI, 1.16–1.86; *p* = 0.001) also had significantly higher odds of abdominal obesity than non-smokers. The OR was highest among e-smokers and lowest among c-smokers. In the adjusted model, e-smokers (OR 2.03; 95% CI, 1.16–3.53; *p* = 0.013) and dual smokers (OR 1.74; 95% CI, 1.35–2.24; *p* < 0.001) had significantly higher odds of abdominal obesity than non-smokers.

In comparisons among current smokers, e-smokers had higher odds of abdominal obesity than c-smokers in the crude model (OR 1.46; 95% CI, 0.85–2.49; *p* = 0.169), but the result was not statistically significant. Dual smokers also showed higher odds of abdominal obesity than c-smokers (OR 1.22; 95% CI, 0.95–1.55; *p* = 0.114), but this finding was also not statistically significant (Table 3). In the adjusted model, dual smokers had significantly higher odds of abdominal obesity than c-smokers (OR 1.30; 95% CI, 1.01–1.68; *p* = 0.040). E-smokers also showed higher odds of abdominal obesity than c-smokers (OR 1.52; 95% CI, 0.88–2.63; *p* = 0.133), but this association was not statistically significant.

## 4. Discussion

This cross-sectional study used data from the Korea National Health and Nutrition Examination Survey (KNHANES), a nationally representative survey of the Korean population, and analyzed the associations between smoking status/smoking type and obesity as well as abdominal obesity in a large population sample. A key contribution of this study is that it distinguished conventional cigarette smoking from electronic cigarette use and evaluated differences in obesity-related risk across smoking types. Overall, smoking was associated with a higher prevalence of abdominal obesity compared with non-smoking in adjusted models, and both dual users and electronic cigarette users showed higher point estimates for obesity-related outcomes than conventional cigarette smokers, though these findings are subject to the limitations discussed below.

In the analysis by smoking status, former smokers showed the highest prevalence of obesity and abdominal obesity, whereas non-smokers showed the lowest prevalence, suggesting potential weight gain after smoking cessation. In the regression analyses before adjustment for confounders, both current and former smokers had significantly higher odds of obesity and abdominal obesity than non-smokers. Notably, former smokers showed approximately 1.4-fold higher risks compared with non-smokers, supporting the possibility of metabolic changes and weight gain after cessation. However, after adjusting for major confounders—including sex, age, physical activity, alcohol consumption, hypertension, and diabetes—the associations between smoking status and obesity/abdominal obesity were no longer statistically significant. This finding suggests that post-cessation weight gain does not necessarily translate into generalized obesity or abdominal obesity, and that the relationship between smoking cessation and adiposity is likely influenced by multiple lifestyle and clinical factors. More rigorous study designs and longitudinal follow-up are needed to clarify the causal relationship between smoking cessation and weight gain.

In the analysis by smoking type, electronic cigarette users showed substantially higher odds of obesity and abdominal obesity than non-smokers. Before adjustment for confounders, electronic cigarette users had 2.35-fold higher odds of obesity and 1.75-fold higher odds of abdominal obesity; after adjustment, these associations remained statistically significant (obesity: 1.98-fold; abdominal obesity: 2.03-fold). Dual users also showed higher risks of obesity and abdominal obesity than non-smokers after adjustment (obesity: 1.71-fold; abdominal obesity: 1.74-fold). In comparisons among smokers, electronic cigarette users had 1.81-fold higher odds of obesity than conventional cigarette smokers in the crude model, although most other comparisons did not show statistically significant differences. In contrast, dual users consistently showed higher risks than conventional cigarette smokers, even after adjustment (obesity: 1.42-fold; abdominal obesity: 1.30-fold). Given the small number of exclusive electronic cigarette users in this study (*n* = 57), all findings pertaining to this subgroup should be considered exploratory. The limited sample size resulted in wide confidence intervals, reduced statistical power for detecting moderate effects, and potentially unstable point estimates that are sensitive to individual observations. Although the consistently elevated point estimates among electronic cigarette users are noteworthy—particularly given the corroborating pattern observed among the larger dual user group (*n* = 310)—these preliminary findings require confirmation in future studies with substantially larger e-cigarette user samples before any firm conclusions can be drawn.

Importantly, given the cross-sectional nature of this study, the possibility of reverse causation must be carefully considered. The observed associations between e-cigarette use and higher odds of obesity and abdominal obesity do not necessarily indicate that e-cigarette use contributes to weight gain. Rather, individuals with higher BMI may preferentially initiate or switch to e-cigarette use due to beliefs that vaping may aid in weight control. Prior research has documented that a notable proportion of adult e-cigarette users report vaping specifically for weight loss or weight control, with overweight individuals being significantly more likely to endorse this motivation [22]. Furthermore, weight-control motivations for e-cigarette use appear to be particularly prevalent among individuals with disordered eating behaviors [23], suggesting that body weight concerns may drive e-cigarette uptake. Additionally, obesity is known to influence nicotine pharmacokinetics and metabolism, which may in turn affect smoking behavior and product choice [24]. The cross-sectional design of this study cannot disentangle these potential bidirectional pathways, and therefore, all findings reported herein should be interpreted as cross-sectional associations rather than evidence of causal relationships. Prospective longitudinal studies with repeated assessments of both smoking behavior and anthropometric measures are essential to clarify the temporal sequence and directionality of these relationships.

In addition, the smoking groups in this study differed substantially in age and sex distributions, reflecting the demographic patterns of smoking behavior in the Korean population. Although age and sex were adjusted for in the multivariable models, such structural differences between groups may lead to residual confounding that covariate adjustment alone cannot fully address. Sex-stratified analysis was not feasible due to the very small number of female e-cigarette users (*n* = 9), and propensity score matching was not performed because of concerns that it would further reduce the already limited e-cigarette subgroup, resulting in unstable estimates. Future studies with larger and more demographically balanced samples of e-cigarette users should employ stratified analyses or propensity score methods to more rigorously control for these structural confounders.

Several biological mechanisms have been proposed that may be relevant to the observed associations between smoking behavior and obesity. At first glance, the positive associations between smoking and obesity observed in this study may appear to contradict the well-established acute effects of nicotine, which include appetite suppression, increased energy expenditure through sympathetic activation, and modest weight reduction in short-term experimental studies [25]. However, the relationship between nicotine exposure and body composition is complex and likely depends on the chronicity and magnitude of exposure as well as non-nicotine constituents of tobacco and e-cigarette products. Critically, while acute nicotine exposure may suppress appetite, chronic exposure has been shown to promote insulin resistance through sustained elevations in catecholamines and cortisol levels [26], and to alter adipose tissue metabolism in ways that favor visceral fat accumulation even when overall body weight does not increase substantially [27]. This phenomenon—sometimes referred to as the ‘smoker’s paradox’—has been documented in epidemiological studies showing that heavy smokers often exhibit greater abdominal obesity despite lower overall BMI compared with non-smokers or light smokers [28]. Additionally, the dose–response relationship between smoking and adiposity appears to be non-linear: while light smoking may be associated with lower body weight, heavy smoking and greater cumulative nicotine exposure have been linked to metabolic dysregulation and preferential abdominal fat deposition [25,28]. The findings of the present study—in which current smoking was significantly associated with abdominal obesity but not with general obesity after covariate adjustment—are consistent with this mechanistic framework.

It should be noted that waist circumference, the measure used to define abdominal obesity in this study, is highly correlated with BMI in most populations [29]. Consequently, the observed associations for abdominal obesity may partly reflect overall adiposity rather than a distinct central fat distribution pattern. However, the finding that current smoking was significantly associated with abdominal obesity but not with general obesity after covariate adjustment suggests that WC may capture aspects of adiposity beyond what BMI alone reflects. Alternative allometric indices such as A Body Shape Index (ABSI), which adjusts waist circumference for its expected scaling relationship with BMI and height, have been shown to provide a more specific measure of abdominal adiposity independent of overall body size [29,30]. Notably, Nagayama et al. (2023) [31] demonstrated that cumulative cigarette consumption was associated with ABSI-assessed abdominal obesity mediated through cardio-ankle vascular index, further supporting the relevance of allometric indices in smoking research. Future studies should employ ABSI or similar indices to more precisely characterize the relationship between smoking patterns—including electronic cigarette use—and central fat distribution [31].

In addition to nicotine-related mechanisms, electronic cigarettes expose users to a variety of aerosolized constituents, including carbonyl compounds (formaldehyde, acrolein), flavoring agents, and solvents. These substances may induce oxidative stress and inflammatory responses and have been implicated in disturbances of glucose metabolism and lipid regulation [32,33,34,35]. Experimental studies have also shown that nicotine-free e-liquids can adversely affect hepatic lipid metabolism and glucose homeostasis, suggesting that components other than nicotine may contribute to metabolic alterations [36]. Together, these mechanisms may be relevant to understanding the observed associations between electronic cigarette use and higher odds of obesity and abdominal obesity in the present study [37,38]. Although prior studies, including the present one, suggest links between e-cigarette use and metabolic abnormalities, the underlying mechanisms remain unclear, and the specific constituents responsible have not been identified. Future research should elucidate constituent-specific toxicological mechanisms and confirm causal relationships through long-term longitudinal studies.

In this study, dual users had higher risks of obesity and abdominal obesity than conventional cigarette smokers. This may be explained by increased total exposure to nicotine and other toxicants from the concurrent use of both products [39]. Prior research has also reported that dual users tend to have greater daily energy intake, higher levels of stress and depressive symptoms, and less healthy dietary patterns compared with single-product users [40]. These behavioral characteristics may contribute to the higher obesity prevalence observed among dual users. Additionally, dual users have been reported to exhibit adverse cardiometabolic profiles—including elevated triglycerides and lower HDL cholesterol—although these metabolic syndrome components are conceptually distinct from obesity per se and may represent either consequences of or parallel pathways alongside excess adiposity [40]. Therefore, e-cigarette use and dual use should not be assumed to be metabolically neutral substitute smoking behaviors. However, whether these associations reflect causal effects or confounding and selection processes remains to be determined through future longitudinal research.

It should also be acknowledged that several important determinants of obesity were not included as covariates in this study. Dietary intake—including total caloric intake, macronutrient composition, and dietary quality—is a well-established determinant of obesity that may differ across smoking groups. Smokers have been reported to have dietary patterns characterized by lower fruit and vegetable intake, higher consumption of energy-dense foods, and greater alcohol consumption compared with non-smokers, and these dietary differences may partly account for the observed associations. Recent evidence has further highlighted the role of dietary quality, particularly patterns characterized by higher intake of ultra-processed foods, in relation to metabolic parameters and health outcomes [41], underscoring the importance of dietary assessment in studies examining obesity-related associations. However, dietary variables in KNHANES are based on a single-day 24 h recall and may not accurately capture habitual dietary patterns. Moreover, dietary behavior may function as a mediator rather than a confounder in the smoking–obesity relationship, as smoking is known to influence appetite, taste preferences, and food choices. Similarly, other unmeasured variables—including sleep duration, sedentary time, occupational status, and medication use—may confound or mediate the observed associations and represent additional sources of residual confounding. The inability to account for these factors limits the precision of the estimated associations and should be considered when interpreting the findings.

Despite its strengths—namely, the use of nationally representative KNHANES data and a large sample size—this study has several limitations. First, due to the cross-sectional design, causal relationships between smoking status/type and obesity or abdominal obesity cannot be established. Because the relationship between smoking and obesity is likely bidirectional—smoking behaviors may influence body weight, and conversely, body weight and weight-control motivations may influence smoking product choice—longitudinal studies with repeated measurements are essential to establish the temporal sequence and causal direction of these associations. Second, smoking status and smoking type were classified using both self-reported questionnaires and urinary cotinine levels; however, self-report data may be subject to underreporting or overreporting. Although cotinine concentrations were incorporated to address the potential for misreporting—particularly in East Asian populations where false reporting among self-identified non-smokers may be relatively common—measurement error cannot be completely ruled out. Additionally, the use of a single urinary cotinine cut-off (100 ng/mL) to verify self-reported smoking status may introduce misclassification bias. Elevated cotinine levels may result from sources other than active smoking, including second-hand smoke exposure, use of heated tobacco products, or nicotine replacement therapy. While second-hand smoke exposure typically produces cotinine levels well below the 100 ng/mL threshold, and the prevalence of heated tobacco products and nicotine replacement therapy in Korea during 2016–2018 was relatively low, the possibility of residual misclassification cannot be excluded. Non-differential misclassification would be expected to attenuate the observed associations, whereas differential misclassification could bias results in either direction. Future studies should incorporate more detailed questions on all nicotine-containing product use, including heated tobacco products and nicotine replacement therapy, to minimize classification error. Another major limitation is the small number of exclusive electronic cigarette users (*n* = 57), which substantially limits the statistical power and precision of the estimates for this subgroup. Post hoc power analysis indicates that our study had sufficient power to detect only large effects (OR ≥ 2.0) in comparisons between e-cigarette users and non-smokers, and considerably less power for comparisons among current smoker subgroups. The wide confidence intervals and the sensitivity of point estimates to individual observations in this small subgroup mean that all findings related to exclusive e-cigarette users should be regarded as exploratory and hypothesis-generating. Future studies utilizing more recent survey waves—in which e-cigarette prevalence has increased substantially—are needed to provide adequately powered analyses and to confirm or refute the patterns observed in the present study.

It should also be noted that the substantial differences in age and sex distributions across smoking groups—which reflect the demographic epidemiology of smoking in Korea—may contribute to residual confounding despite covariate adjustment. This structural imbalance should be considered when interpreting the magnitude of the observed associations.

Moreover, several important determinants of obesity—including dietary intake, sleep duration, sedentary time, occupational status, and medication use—were not included as covariates. The omission of these variables, particularly dietary factors that are known to differ between smokers and non-smokers, represents a source of residual confounding that may affect the estimated associations.

The complex sampling design of KNHANES—including sampling weights, stratification, and clustering—was not incorporated into the statistical analyses. As a consequence, the standard errors of the estimated odds ratios may be underestimated, potentially resulting in confidence intervals that are narrower than those that would be obtained under design-based analysis. While odds ratio point estimates from logistic regression are generally considered robust to the omission of survey weights, associations with borderline statistical significance—particularly those involving the small e-cigarette subgroup—should be interpreted with additional caution, as they may not retain significance under properly weighted analysis. Additionally, the descriptive statistics reported in Table 1 and Table 2 (e.g., prevalence estimates, means) may not be nationally representative in the absence of sampling weights. Future studies should apply complex sampling procedures, utilizing the survey weights, strata, and primary sampling units provided in the KNHANES dataset, to ensure that both point estimates and variance estimates appropriately reflect the survey design.

Furthermore, abdominal obesity in this study was defined using waist circumference cut-offs, which are highly correlated with BMI (r ≈ 0.9). This limits the ability to distinguish central adiposity from overall obesity. Although the divergent results between general obesity and abdominal obesity in our adjusted models provide some indirect evidence that WC captures information beyond BMI, the use of allometric indices such as A Body Shape Index (ABSI)—which is designed to be independent of BMI—would allow for a more precise assessment of central fat distribution. Future studies incorporating such indices are warranted to confirm and extend the present findings.

## 5. Conclusions

This cross-sectional study examined the associations between smoking behaviors and obesity as well as abdominal obesity using KNHANES 2016–2018 data, with explicit categorization of electronic cigarette use. Dual users consistently showed higher odds of obesity-related outcomes compared with non-smokers, while findings for exclusive electronic cigarette users (*n* = 57) were exploratory and should be interpreted with considerable caution given the small sample size, wide confidence intervals, cross-sectional design, residual confounding, and the absence of complex sampling weights in the analysis. These findings do not establish e-cigarette use as a risk factor for obesity and should not be used to inform policy recommendations at this stage. Further studies with larger samples, longitudinal designs, and comprehensive confounder adjustment are needed to determine whether the observed associations are causal.

## Figures and Tables

**Table 2:** Distribution of obesity by smoking status.

	General Obesity (BMI ≥ 25 kg/m^2^)		Abdominal Obesity (WC ≥ 90 cm in Men/≥85 cm in Women)	
	Yes (n = 6262)	No (n = 11,628)	Yes (n = 5381)	No (n = 12,509)
Smoking status				
Never smoker	3537 (32.1)	7478 (67.9)	3067 (27.8)	7948 (72.2)
Ex- smoker	1401 (40.0)	2100 (60.0)	1225 (35.0)	2276 (65.0)
Current smoker (total)	1324 (39.2)	2050 (60.8)	1089 (32.3)	2285 (67.7)
*p*-value	<0.001		<0.001	
Smoking type				
Current (c-smoker)	1143 (38.0)	1864 (62.0)	954 (31.7)	2053 (68.3)
Current (e-smoker)	30 (52.6)	27 (47.4)	23 (40.4)	34 (59.6)
Current (dual smoker)	151 (48.7)	159 (51.3)	112 (36.1)	198 (63.9)
*p*-value	<0.001		0.121	

Values are presented as number (proportions).

**Table 3:** Risk of obesity by smoking status, compared to never-smokers.

	General Obesity (BMI ≥ 25 kg/m^2^)		Abdominal Obesity (WC ≥ 90 cm in Men/≥85 cm in Women)	
	Crude OR (95% CI)	Adjusted ^a^ OR (95% CI)	Crude OR (95% CI)	Adjusted OR (95% CI)
Smoking status				
Never-smoker	1.00 (reference)	1.00 (reference)	1.00 (reference)	1.00 (reference)
Ex- smoker	1.41 (1.30–1.53) ***	0.99 (0.89–1.09)	1.40 (1.29–1.51) ***	1.04 (0.93–1.15)
Current smoker (total)	1.37 (1.26–1.48) ***	1.06 (0.96–1.17)	1.24 (1.14–1.34) ***	1.17 (1.05–1.30) **
Smoking types				
Never-smoker	1.00 (reference)	1.00 (reference)	1.00 (reference)	1.00 (reference)
Current (c-smoker)	1.30 (1.19–1.41) ***	0.99 (0.89–1.10)	1.20 (1.10–1.31) ***	1.10 (0.98–1.24)
Current (e-smoker)	2.35 (1.40–3.96) **	1.98 (1.16–3.38) *	1.75 (1.03–2.98) *	2.03 (1.16–3.53) *
Current (dual smoker)	2.01 (1.60–2.52) ***	1.71 (1.34–2.17) ***	1.47 (1.16–1.86) **	1.74 (1.35–2.24) ***
Among smoking types				
Current (c-smoker)	1.00 (reference)	1.00 (reference)	1.00 (reference)	1.00 (reference)
Current (e-smoker)	1.81 (1.07–3.06) *	1.65 (0.96–2.81)	1.46 (0.85–2.49)	1.52 (0.88–2.63)
Current (dual smoker)	1.55 (1.23–1.96) ***	1.42 (1.12–1.81) **	1.22 (0.95–1.55)	1.30 (1.01–1.68) *

Values are presented as odds ratio (95% confidence interval). * *p*-value < 0.05, ** *p*-value < 0.01, *** *p*-value < 0.001. ^a^ Adjusted for age, sex, physical activity, alcohol consumption, hypertension, and diabetes.

## Data Availability

Publicly available datasets were analyzed in this study. These data can be found here: Korea National Health and Nutrition Examination Survey (KNHANES), https://knhanes.kdca.go.kr (accessed on 15 December 2025).
